# Comparison of Expression of Secondary Metabolite Biosynthesis Cluster Genes in *Aspergillus flavus*, *A. parasiticus*, and *A. oryzae*

**DOI:** 10.3390/toxins6061916

**Published:** 2014-06-23

**Authors:** Kenneth C. Ehrlich, Brian M. Mack

**Affiliations:** Southern Regional Research Center, Agricultural Research Service, United States Department of Agriculture, New Orleans, LA 70124, USA; E-Mail: brian.mack@ars.usda.gov

**Keywords:** sclerotial morphotypes, gene transcription, non-ribosomal peptide synthase, polyketide synthase, RNA-seq

## Abstract

Fifty six secondary metabolite biosynthesis gene clusters are predicted to be in the *Aspergillus flavus* genome. In spite of this, the biosyntheses of only seven metabolites, including the aflatoxins, kojic acid, cyclopiazonic acid and aflatrem, have been assigned to a particular gene cluster. We used RNA-seq to compare expression of secondary metabolite genes in gene clusters for the closely related fungi *A. parasiticus*, *A. oryzae*, and *A. flavus* S and L sclerotial morphotypes. The data help to refine the identification of probable functional gene clusters within these species. Our results suggest that *A. flavus*, a prevalent contaminant of maize, cottonseed, peanuts and tree nuts, is capable of producing metabolites which, besides aflatoxin, could be an underappreciated contributor to its toxicity.

## 1. Introduction

Biosynthesis of many fungal secondary metabolites, including mycotoxins, typically requires enzymes encoded by sets of clustered genes [1]. With the availability of full genome sequences, genes can be associated with secondary metabolite biosynthesis by use of the software program SMURF [2]. This program allows automated search of the genome to identify sets of contiguous genes that include a “backbone” gene encoding a protein required for biosynthesis of a metabolite precursor [3], a transcription factor for regulation of gene expression, oxidases or reductases for modification of the metabolite precursor and transporters for export or for moving the metabolite to vacuoles or vesicles within the cell [3,4]. For secondary metabolite formation, typical backbone enzymes include non-ribosomal peptide synthases (NRPSs), polyketide synthases (PKSs) [5,6] or geranylgeranyl pyrophosphate synthases (GGPSs) [7] for one or more of the biosynthesis steps. Also, characteristic of some NRPS-derived metabolites is a step involving tryptophan prenylation, which is catalyzed by a cluster-associated dimethylallyltryptophan synthase (DMATS) [8]. The ability of fungi to co-ordinately regulate transcription of clustered genes usually depends on a single sequence-specific DNA-binding protein of the Zn_2_Cys_6_-type unique to a given cluster [9]. Expression of genes controlled by such transcription factors should define the boundaries for the gene cluster [10]. A method that combined SMURF with microarray expression analysis was recently described that also could help to better define the cluster boundaries for genes in secondary metabolite biosynthesis clusters [11].

In the present study expression analysis by RNA-seq was performed on two sclerotial size variants of *A. flavus* (called S and L strains) and the non-aflatoxigenic variant, *A. oryzae*. These *A. flavus* variants are morphologically and phylogenetically distinct [12]. Analysis was also done on *A. parasiticus*, a close relative of *A. flavus* that produces G- in addition to B-aflatoxins. Although RNA-seq data were available for isolates of an *A. flavus* L strain and *A. oryzae* [13,14,15], they were not available for an S strain *A. flavus* or for *A. parasiticus*. The comparison of RNA-seq data described in this paper evaluates the potential of these fungi to produce secondary metabolites when grown on a typical fungal growth medium. Such identification is the first step for rational assignment of a biosynthetic gene cluster to production of a specific metabolite.

## 2. Results and Discussion

### 2.1. Types of Backbone Genes

The gene clusters for secondary metabolism in *A. flavus* NRRL3357 previously identified by SMURF [16] were used for identification and annotation of homologous clusters in the related species: *A. parasiticus*, two variant *A. flavus* S strain isolates and *A. oryzae*. Putative backbone genes for gene clusters identified in *A. flavus* NRRL3357 are given in Table 1, Table 2 and Table 3. The PKS-encoding backbone genes in Table 1 are arranged by types of proteins predicted to be produced by these genes. Those encoding polyketide synthases with reducing domains are distinguished from those encoding proteins that lack such domains. The NRPS genes are arranged in Table 2 by those predicted to encode proteins with repeated condensation (C) domains and those predicted to encode proteins with single or no C domains. For both types of secondary metabolite, putative PKSs and NRPSs with only a single, or at most two, catalytic domains are listed separately. Genes for clusters 23 and 55 are predicted to encode a single polypeptide containing both PKS and NRPS catalytic domains. In Table 1 and Table 2 transcription factors associated with the putative gene clusters are listed separately. Only some of the gene clusters contain transcription factors within the putative cluster [10]. Gene clusters containing the biosynthetic enzymes for production of GGPSs and DMATSs are listed in Table 3. One secondary metabolite whose biosynthesis has recently been studied, kojic acid, is derived from glucose [17]. Because of this difference in biosynthesis it is not shown in these lists or in Table S1.

**Table 1:** Putative polyketide synthase backbone genes in SMURF-identified secondary metabolite clusters in *A. flavus.*

Cluster Number	Type		*A. flavus* NRRL3357		*A. flavus* AF70		*A oryzae* RIB40		*A. parasiticus* BN9		Transcription Factor(s) in AF-3357 Cluster
	aa ^a^	Domains	Gene	RPKM ^c^	Gene	RPKM	Gene	RPKM	Gene	RPKM	
	**Reducing PKS**										
1	2432	KS-AT-DH-MT-PP ^b^	AFLA_002900	**2.0**	3.m000841	**1.9**	AO090102000166	**4.6**	14.m004661	**5.2**	not found
17	2895	KS-AT-DH-MT-ER-KR-NADB/TE	AFLA_053870	0.7	76.m000261	0.5	AO090009000071	**1.1**	9.m006082	0.9	AFLA_053760
20	2355	KS-AT-DH-MT-KR-ER-KR-PP	AFLA_062820	**1.1**	310.m000108	**12.0**	AO090701000826	**2.5**	3.m008254	**18.0**	AFLA-62960
23	2462	KS-AT-DH-MT-KR-PP	AFLA_066980	**3.7**	401.m000099	**2.0**	AO090001000293	**2.2**	not found		AFLA-066830,066960,066900
40	2137	KS-AT-DH-PP	AFLA_112840	0.5	148.m000228	0.5	AO090023000877	0.6	not found		AFLA-112830
46	2460	KS-AT-DH-MT-ER-KR-PP	AFLA_118940	0.1	4.m000822	0.0	AO090010000402	0.1	11.m006552	**19.1**	not found
50	2505	KS-AT-DH-MT-ER-KR	AFLA_126710	**1.2**	217.m000143	0.0	AO090038000210	**1.1**	6.m007393	**2.0**	AFLA-126910
52	2591	KS-AT-DH-MT-ER-TE-PP	AFLA_128060	0.3	182.m000166	0.7	AO090001000506	**1.8**	6.m007542	**9.2**	AFLA-128150,128160
	**Non-reducing PKS**										
5	2141	KS-AT-PP-PP-TE	AFLA_006170	**1.3**	29.m000459	**1.1**	AO090102000545	**1.2**	14.m004338	**42.8**	AFLA-006240
20	2245	KS-AT-PP-TE	AFLA_062860	**2.1**	310.m000104	**34.9**	AO090701000831	**6.5**	3.m008250	**38.9**	AFLA-062960
27	2045	KS-AT-PP-TE	AFLA_082150	0.3	8.m000609	**1.6**	AO090005000961	0.0	3.m008687	**1.2**	AFLA-082140
33	947	KS-AT	AFLA_096770	0.0	513.m000031	0.0	AO090113000209	0.0	not found ^d^		not found
38	2475	KS-AT-MT-MT-KR	AFLA_105450	**4.2**	655.m000042	0.8	not found		not found		not found
39	1751	KS-AT-PP	AFLA_108550	0.0	152.m000223	0.0	AO090023000444	0.2	16.m004060	0.1	not found
41	1120	KS-AT-KR-PP	AFLA_114820	**2.4**	255.m000114	0.8	AO090206000074	**1.5**	21.m001060	**1.6**	not found
42	2104	KS-AT-PP-TE	AFLA_116220	0.0	4.m000888	0.1	AO090010000048	0.0	11.m006280	0.1	AFLA-116230
44	2580	KS-AT-PP-MT-TE	AFLA_116890	0.2	4.m000824	**1.0**	AO090010000114	0.3	11.m006344	0.0	AFLA-116880
46	2253	KS-AT-PP-MT	AFLA_118960	0.1	39.m000415	0.2	AO090010000404	0.2	11.m006554	**21.8**	not found
51	2586	KS-AT-PP-TE	AFLA_127090	0.2	268.m000166	0.2	AO090001000402	**1.3**	6.m007438	**3.5**	AFLA-126990
54	2109	KS-AT-PP	AFLA_139410	**197.0**	210.m000122	**1.4**	AO090026000009	**4.2**	5.m007293	**194.0**	AFLA-139360
	**Short PKS**										
7	396	KS-PP	AFLA_009140	0.4	19.m000416	**1.0**	AO090103000313	0.2	15.m004154	0.0	not found
8	396	KS-AT-DH-MT	AFLA_010000	0.4	365.m000072	**1.4**	AO090103000224	0.8	not found		not found
17	327	DH	AFLA_053780	0.0	169.m000208	0.0	AO090009000078	0.0	not found		AFLA-053760
26	207	TE-PP	AFLA_079360	0.0	803.m000023	0.0	AO090005000687	0.0	8.m006320	0.0	AFLA-079320
36	689	KS	AFLA_104210	0.0	201.m000178	0.1	not found		not found		not found
36	301	KS	AFLA_104240	**2.6**	201.m000181	0.2	not found		not found		not found
36	696	ER	AFLA_104250	**5.2**	not found		not found		not found		AFLA-104220
43	413	KR-PP	AFLA_116500	0.0	4.m000863	0.0	not found		not found		not found
49	426	KR-PP	AFLA_125630	0.0	not found		not found		6.m007262	0.0	not found
49	708	AT-DH	AFLA_125640	0.0	376.m000099	0.0	AO090038000086	0.0	not found		AFLA-125590

Notes: ^a^ aa-length in amino acids; ^b^ Domains: KS-ketosynthase; AT-acyltransferase; DH-dehydratase; ER-enoyl reductase; KR-ketoreductase; PP-Phosphopantetheine attachment site; MT-methyltransferase; TE-thioesterase; ^c^ RPKM values are from cultures grown on potato dextrose agar medium in the dark for two days. RPKM vaues >1 are shown in bold font; ^d^ not found: BLASTN search against the *A. flavus* NRRL3357 genome produced no alignments with E value below 1e-10 and a percent identity above 80%.

**Table 2:** Putative non-ribosomal peptide synthase backbone genes in SMURF-identified secondary metabolite clusters in *A. flavus.*

Cluster Number	Type		*A. flavus* NRRL3357		*A. flavus* AF70		*A. oryzae* RIB40		*A. parasiticus* BN9		Transcription factor in AF-3357 cluster
	aa ^a^	Domains ^b^	gene	RPKM ^c^	gene	RPKM	gene	RPKM	gene	RPKM	
	**Large NRPSs-di, tri,tetra peptide types ^a^**										
3	5011	C-A-T-C-C-A-T-C-A-T-C-A-T	AFLA_004450	**2.3**	11.m000536	0.2	AO090102000338	**2.9**	14.m004504	**2.6**	AFLA_005290
4	2621	C-A-T-C-A-T-C	AFLA_005440	**1.0**	507.m000046	0.2	AO090102000465	0.1	not found		AFLA_005520
6	5209	A-C-C-A-T-C-A-T-C-A-T-C-C	AFLA_008770	0.1	19.m000449	0.0	AO090103000355	0.0	15.m004127	0.4	
9	7763	A-C-A-C-C-A-T-C-A-C-A-M-C-A-R	AFLA_010580	**1.4**	115.m000177	**2.6**	not found		15.m004289	0.5	
9	2100	A-T-C-A-T-C	AFLA_010620	0.9	115.m000173	0.6	AO090103000167	**7.8**	15.m004294	0.6	
13	2975	A-T-C-A-T-C-A	AFLA_038600	0.2	124.m000181	**2.4**	AO090011000043	**1.9**	4.m008917	**4.2**	
21	2074	A-T-C-A-T-Cpartial	AFLA_064240	**16.3**	62.m000377	**1.3**	AO090001000009	**1.7**	12.m006349	**15.8**	AFLA_064370
22	5326	A-T-C-A-T-C-A-C-A-T-C-A-T-C	AFLA_066720	0.3	123.m000188	0.1	AO090001000262	0.5	not found		
24	5186	A-T-C-C-A-T-C-A-T-C-C-T-C	AFLA_069330	**17.2**	100.m000228	**22.0**	AO090038000390	**2.1**	18.m003390	**41.1**	
		**Single A-domains-A-C**									
8	1626	T-C-A-T-R	AFLA_010010	**1.1**	not found		not found		15.m004242	0.0	
8	1338	A-T-C	AFLA_010020	**1.8**	579.m000030	**2.1**	AO090103000223	**2.2**	15.m004243	0.6	
34	1225	A-T-C	AFLA_100340	0.0	not found ^d^		not found		6.m007273	0.8	AFLA_100300
53	1071	A-T-C	AFLA_135490	0.1	not found		not found		not found		
21	1621	T-C-A-C	AFLA_064560	0.5	62.m000409	0.1	AO090001000043	**6.8**	12.m006318	**2.0**	
30	1735	A-T-C-T-C	AFLA_090200	0.0	215.m000247	0.1	AO090120000024	0.0	7.m007260	0.1	
		**Single A-domains-A-T**									
11	1021	A-T-SDR_e1	AFLA_023020	0.1	20.m000466	0.0	AO090003001545	0.0	1.m012869	**1.3**	AFLA_023040
12	1011	A-T-R	AFLA_028720	**1.5**	242.m000170	0.1	AO090003000945	0.2	1.m013429	**5.2**	
18	1251	A-T-R-gntK	AFLA_054270	0.1	307.m000171	0.0	AO090009000033	0.3	9.m006043	0.0	AFLA_054310
25	1008	A-TE	AFLA_070920	0.1	304.m000110	0.0	AO090038000550	0.0	19.m002212	**1.7**	
26	957	A-T-R	AFLA_079380	0.9	333.m000120	**5.4**	AO090005000688	**8.6**	8.m006319	**1.8**	
26	1278	A-T-SDR_e1	AFLA_079400	**5.2**	333.m000118	**7.4**	AO090005000690	**16.2**	8.m006317	**20.9**	AFLA_079320
37	1055	A-R	AFLA_105190	0.9	348.m000125	0.6	AO090023000082	**6.0**	17.m003740	**13.7**	AFLA_118300
45	1048	C-A-T-R	AFLA_118440	0.2	137.m000247	0.0	AO090010000349	0.0	11.m006507	0.0	
47	1043	A-T-R	AFLA_119110	0.1	395.m000106	0.1	AO090010000426	0.0	11.m006588	0.0	
35	1042	A-T-SDR-e1	AFLA_101700	0.8	1.m000978	**1.1**	AO090020000240	0.7	10.m006579	0.0	
48	1007	A-T-SDR-e1	AFLA_121520	0.6	not found		not found		not found		
		**Short NRPSs**									
7	611	A-T-epimerase	AFLA_009120	0.5	19.m000418	**4.9**	AO090103000316	0.4	not found		
28	396	T-C	AFLA_082480	0.0	not found		AO090005000993	0.0	not found		
33	163	T	AFLA_096700	0.0	36.m000454*	0.0	AO090113000200	0.0	7.m006639	0.0	
33	317	C	AFLA_096710	0.0	not found		AO090113000201	0.5	7.m006638	0.0	
		**Hybrid PKS/NRPSs**									
23	3946	KS-AT-DH-M-KR-T-C-A-T-T-R	AFLA_066840	0.7	123.m000175	0.7	AO090001000277	0.9	12.m006079	**2.8**	AFLA_066830,066860,066900
55	3851	KS-AT-DH-M-KR-T-C-A-T-R	AFLA_139490	**6.0**	210.m000130	**2.7**	AO090026000001	0.5	5.m007288	**1.2**	AFLA_139500

Notes: ^a^ length in amino acids; ^b^ Domain abbreviations: A-adenylation; C-condensation; T-thiolation; M-methyltransferase; R-reductase; T-thioesterase; SDR_e1-short-chain dehydrogenases/reductases; gntK-gluconokinase; KS-ketosynthase; AT-acytransferase; DH-dehydratase; KR-ketoreductase; ^c^ RPKM values are from cultures grown on potato dextrose agar medium in the dark for two days. RPKM vaues >1 are shown in bold font; ^d^ not found: BLASTN search did not give hits with E value below 1e-10 and a percent identity above 80%.

**Table 3:** Putative GGPS or DMATS backbone genes in SMURF-identified secondary metabolite clusters in *A. flavus* NRRL3357.

Cluster Number	Type	*A. flavus* NRRL3357		*A. flavus* AF70		*A. flavus* CA14	*A. oryzae* RIB40		*A. parasiticus* BN9		Transcription factor in cluster
		Gene	RPKM ^a^	Gene	RPKM ^a^	RPKM ^b^	Gene	RPKM ^a^	Gene	RPKM ^a^	
2	DMATS	AFLA_004300	0.0	11.m000553	0.0	0.1	AO090102000322	0.0	14.m004523	0.0	AFLA_004280
15	DMATS	AFLA_045490	0.0	24.m000477	0.2	**104.3**	AO090011000738	0.0	4.m008255	0.0	
19	DMATS	AFLA_060680	**68.8**	165.m000196	**37.2**	0.6	AO090701000600	**134.8**	3.m008454	**19.7**	
22	GGPS	AFLA_066780	0.6	123.m000181	0.5	0.3	AO090001000268	**1.3**	not found		
32	GGPS	AFLA_096390	0.0	36.m000482	0.0	**129.4**	AO090113000171	0.0	7.m006673	0.0	AFLA_096370
37	GGPS	AFLA_105050	**10.0**	50.m000356	**1.0**	0.4	AO090023000070	**13.7**	17.m003755	**1.6**	
43	DMATS	AFLA_116600	**2.6**	4.m000853	0.5	**1.0**	AO090010000082	**17.3**	11.m006315	0.4	

Notes: ^a^ RPKM values were determined for cultures grown for 40 h on PDA medium; ^b^ RPKM values were determined for cultures grown for 168 h; CA42 is an S-strain isolate similar to AF70.

**Table 4:** Secondary metabolite backbone genes not assigned to *A. flavus* SMURF-identified gene clusters.

Type		*A. flavus* NRRL3357		*A. flavus* AF70 gene		*A. oryzae* RIB40		*A. parasiticus* BN9	
aa ^a^	Domains ^b^	Gene	RPKM ^c^	Gene	RPKM	Gene	RPKM	Gene	RPKM
**Polyketide synthase**									
2595	KS-AT-DH-M-ER-PP	AFLA_005320	**3.4**	not found		not found		not found	
1481	KS-DH-ER-ER-KR-PP	AFLA_038310	**1.7**	186.m000172	0.4	AO090011000015	0.6	4.m008944	0.9
2895	KS-AT-DH-M-ER-NADP-SDR_e1	AFLA_053870	0.7	76.m000261	0.5	AO090009000071	**1.1**	9.m006082	0.9
2574	KS-A-DH-MT-ER-ER-FabG-PP	AFLA_054090	0.0	76.m000280	0.0	AO090009000052	0.0	9.m006060	0.1
1254	KS-AT-PP	AFLA_060020	0.1	407.m000089	**2.9**	AO090701000530	**4.7**	13.m005208	0.2
2581	KS-AT-DH-M-ER-ER-KR-PP	AFLA_080490	0.0	34.m000394	0.0	AO090005000798	0.0	8.m006222	0.0
2390	KS-AT-DH-ER-KR-FabG-PP	AFLA_137870	**2.7**	35.m000427	0.5	AO090026000149	**4.3**	5.m007445	**4.0**
2569	KS-AT-DH-M-ER-KR	not found^d^		220.m000181	0.0	not found		not found	
2609	KS-AT-M-ER-KR	not found		59.m000347	0.0	not found		not found	
2648	KR-KS-AT-PP-TE	not found		71.m000353	0.0	not found		9.m006148	0.0
2122	KS-AT-PP-PP	not found		not found		not found		4.m008736	0.0
2482	KS-AT-DH-M-ER-KR-PP	not found		not found		not found		3.m008413	0.0
2441	KS-AT-DH-M-ER-KR-PP	not found		not found		not found		2.m009777	0.0
**Non-ribosomal peptide synthase**									
1000	A-T-TE	AFLA_017840	3.4	53.m000365	2.4	not found		2.m009629	14.8
950	A-T-NADB	AFLA_041610	0.1	75.m000340	0.0	AO090011000328	0.1	4.m008622	0.5
677	A-T-TE	AFLA_082050	0.0	8.m000601	0.1	AO090005000952	0.0	3.m008680	0.0
4760	A-C-A-C-A-C-C-C	AFLA_109430	**2.7**	119.m000213	0.2	AO090023000528	**5.6**	16.m003972	**1.1**
1048	A-TE	AFLA_118440	0.2	137.m000247	0.0	AO090010000349	0.0	11.m006507	0.0
690	A-SDR_e1	AFLA_119820	**2.2**	2.m000879	0.3	AO090010000498	**1.6**	11.m006651	0.0
1068	CaiC-A-TE	AFLA_128170	**0.4**	182.m000155	**1.9**	AO090001000516	**1.8**	6.m007553	0.0
2465	A-T-C-T-C-TE-T-C	AFLA_139670	0.0	not found		not found		12.m006359	0.1
3987	A-C-A-M-C-A-TE	not found		not found		not found		6.m007274	0.0
476	A	not found		not found		not found		4.m008952	0.0
1015	A-T-C	not found		not found		not found		4.m008858	0.0
986	A-T-R	not found		not found		not found		6.m007176	0.0
1338	A-T-C	not found		not found		not found		5.m007834	0.0
1848	A	not found		281.m000120		not found		6.m007331	0.0
**Dimethylallyltryptophan synthase**									
435	DMATS	AFLA_083250	0.2	118.m000246	**1.5**	AO090005001079	0.2	7.m006674	0.0
290	DMATS	AFLA_084080	0.0	83.m000321	0.0	AO090005001168	0.0	3.m008454	0.0
354	DMATS	AFLA_090190	0.0	215.m000248	0.0	AO090120000023	0.0	3.m008862	0.0
435	DMATS	AFLA_083250	0.2	not found	**1.5**	not found	0.2	3.m008784	0.0
474	DMATS	not found		not found		not found		14.m004413	0.0
**Geranylgeranylpyrophosphate synthase**									
389	GGPS	AFLA_018310	**18.5**	357.m000134	**8.3**	AO090012000573	**16.0**	2.m009580	**31.8**
444	GGPS	AFLA_038720	**6.9**	248.m000185	0.5	AO090011000054	**18.4**	2.m009476	**3.4**
369	GGPS	AFLA_053620	**2.1**	169.m000225	**3.3**	AO090009000093	**6.2**	7.m007224	**5.7**
728	GGPS	AFLA_056820	**23.9**	235.m000158	**9.6**	not found		4.m008907	**29.7**
387	GGPS	AFLA_066780	0.6	not found		AO090001000268	**1.3**	not found	
271	GGPS	AFLA_070370	0.0	138.m000238	0.0	not found		19.m002158	0.0
497	GGPS	AFLA_070380	0.0	138.m000238	0.2	AO090038000495	0.0	13.m004891	0.0
315	GGPS	AFLA_073740	**9.7**	369.m000106	**37.8**	AO090005000132	**13.0**	8.m006850	**51.0**
273	GGPS	AFLA_090640	0.0	143.m000255	0.7	AO090120000064	0.0	4.m008906	**2.3**

Notes: ^a^ aaa-length in amino acids; ^b^ Domains: KS-ketosynthase; AT-acyltransferase; DH-dehydratase; ER-enoyl reductase; KR-ketoreductase; PP-Phosphopantetheine attachment site; M-methyltransferase; TE-thioesterase. A-adenylation; C-condensation; T-thiolation; R-reductase; SDR_e1-short-chain dehydrogenases/reductase; FabG-3-oxoacyl-(acyl-carrier-protein) reductase; CaiC-carnitine CoA ligase; NADB-NAD-binding; ^c^ RPKM values were determined for cultures grown for 40 h on PDA medium; ^d^ not found-tBlastX search did not give hits with E value = 0.

### 2.2. Comparison of Putative Secondary Metabolite Clusters from A. oryzae, A. flavus S and L morphotype Isolates and A. parasiticus

Table 1, Table 2 and Table 3 compare secondary metabolite backbone genes in the SMURF-identified gene clusters in *A. flavus* NRRL3357 [16] with homologs in the other isolates. Homologs were determined by reciprocal best hit BLASTN search against the Genbank database for *A. flavus* NRRL3357. Additionally, we selected only the BLAST hits that had an expect (E) value below 1e-10 and a percent identity above 80%. By this criterion, the PKSs encoded by genes in clusters 23, 33, 36, 38, 40, 43, and 49 were not identified in the *A. parasiticus* genome and PKSs in clusters 36 and 43 were not identified in *A. oryzae* (Table 1). Of the NRPS clusters, *A. flavus* backbone genes in clusters 4, 7, 22, 28, 48 and 53 in *A. parasiticus*, in 34, 48, and 53 in AF70, and in 9 and 48 in *A. oryzae* were not identified in the genomes of these isolates (Table 2). The GGPS gene associated with cluster 22 was not identified in *A. parasiticus* (Table 3). NRPS, PKS, DMATS and GGPS genes that were not recognized by SMURF as being in a secondary metabolite gene cluster in *A. flavus* NRRL3357 are shown in Table 4 with their putative homologs in the other isolates. Some of these genes may be in, as yet, unrecognized secondary metabolite biosynthesis clusters. While many of these genes are present in all isolates, seven are found only in *A. parasiticus*. These may represent genes encoding biosynthesis of metabolites unique to *A. parasiticus*. Supplementary Table S2 lists the genes surrounding some of these backbone genes.

### 2.3. RNA-seq Analyses

For RNA-seq analysis we grew the fungi on PDA, a medium previously found to stimulate production of a wide variety of fungal secondary metabolites, including the aflatoxins [18], to determine which backbone genes clusters are actively transcribed. RNA-seq RPKM values are given in Table 1, Table 2, Table 3 and Table 4 and in Supplemental Tables S1 and Supplemental Tables S2. For the purpose of comparison of these data, we consider that an RPKM value less than 1 represents, at most, only a low level of expression, whereas an RPKM value greater than 1 represents detectable expression. Based on these criteria, the RPKM values shown in Table 1 suggest that under our growth conditions, only half of the 29 PKSs and 26 NRPSs for any one isolate can be considered to be expressed and in some cases, the backbone genes that were expressed in the different isolates had markedly different RPKM values. The most prominent differences were found for PKSs in clusters 5, 38, 46, and 52 (Table 1) and for NRPSs in clusters 21, 26, 37, and 55 (Table 2). Some of the backbone genes not previously assigned to gene clusters (Table 4) have RPKM values >1 and potentially could express genes that encode secondary metabolite biosynthesis enzymes. *A. flavus* CA42, an S strain isolate similar to AF70 (shown only in Table 3 and Table S1) gives much higher RPKM values for the PKS genes in clusters 1, 27 and 39, the NRPS genes in clusters 12, 23, 25, 35, 37 and 55, and the DMATS and GGPS genes for aflatrem production in clusters 15 and 32 when grown for 168 h than when grown for only 40 h. At these longer times S strain *A. flavus* produce abundant sclerotia. It is possible that timing of expression for some of the gene clusters is coordinated with sclerotial production and that the associated metabolites accumulate preferentially in sclerotia. To support this conjecture we found, in a separate study, that aflatrem was produced abundantly by both S strain isolates only when sclerotia are formed (Ehrlich and DianaDiMavungu, unpublished results) and under these conditions the genes for the aflatrem biosynthesis (in clusters 15 and 32) were expressed with high RPKM values. Also, the gene for cluster 27 PKS, which was shown to be necessary for most sclerotial pigmentation [19], only is expressed highly in cultures undergoing sclerotial formation (*A. flavus* CA42 in Table S1). Several of the non-reducing PKS genes that are differentially expressed in the different isolates, based on homology to genes in other fungi [20], are predicted to be associated with production of polyketides required for pigment formation, for example, those in clusters 5, 36, 39 and 42. The gene for the DMATS in cluster 19 was expressed at a high RPKM level in most isolates while the GGPS of cluster 37 (an NRPS cluster) was expressed at the highest level in NRRL3357.

These data show that the combination of RNA-seq analysis of secondary metabolite gene expression with SMURF-derived tabulation of putative backbone biosynthetic genes and their clustered common decorating genes is able to provide an accurate way to assess which secondary metabolite biosynthesis gene clusters encode the genes for metabolite production under a given set of growth conditions. However, it is possible that, even if the genes in a cluster are expressed, the resulting protein(s) may not be functional. Most of the PKS and NRPS genes listed in Table 1 and Table 2 as short sequences and which only encode one or two domains of a PKS or NRPS gave no or low RPKM values in our study with the exception of the putative ketosynthase and enoyl reductase genes in cluster 36, the ketosynthase genes in clusters 7 and 8, and the epimerase gene in cluster 7 (Table 1 and Table 2). While these backbone genes are annotated in the databases as PKS- or NRPS-encoding genes, usually such genes are quite large and encode multifunctional enzymes [5,6]. It is possible that for some of these clusters the genes were not annotated correctly in the database and that neighboring sequence should be included in establishing the identity of these protein-coding regions. However, given the lack of expression of most of these genes and their abnormal size, it is likely that such gene clusters, by themselves, do not encode proteins involved in formation of a secondary metabolite.

To prove that a gene cluster actually is involved in biosynthesis of a particular metabolite produced by these closely related *Aspergilli* (for a list of metabolites known to be produced by the isolates examined, see Supplemental Table S3), gene knockout and add back experiments must be done to show that the knockout mutant loses and regains, respectively, the ability to produce the metabolite. Such knockout gene experiments have been done, so far, to confirm the roles of clusters 15 and 32 in production of aflatrem [7], clusters 35 and 48 in production of two related piperazines [21], cluster 27 in production of asparasone [19], cluster 54 in production of aflatoxin [22], and cluster 55 [8] in production of cyclopiazonic acid. In studies of *A. flavus*, *A. oryzae* and *A. parasiticus*, about 20 different classes of metabolites have been isolated from culture extracts [18,23]. Because the types of backbone biosynthetic enzymes often indicate the probable type of metabolite that can be produced based on the catalytic properties of the main PKS or NRPS in the cluster [24,25] the RNA-seq data are consistent with production of about 20 different classes of metabolites. Since many of the putative backbone genes listed in Table 1, Table 2, Table 3 and Table 4 were not expressed, it is possible that these inactive clusters could become active under different growth conditions. In the present study only one growth condition (PDA) was used. It was previously found that gene activity can be induced by association of fungi with the proper microbial or nutritional environment or by artificial alteration of the chromatin state of the genes in the cluster [24,26,27]. The availability of RNA-seq data should improve the chances of being able to select a secondary metabolite backbone gene, that when disrupted, will actually result in loss of production of a specific metabolite.

## 3. Experimental Section

### 3.1. Aspergillus Species Chosen for Comparison

S strain *A. flavus* isolate, CA42, was obtained from almonds in California [28] and AF70 from cotton in Arizona [29]. *A. parasiticus* BN009E (BN9) was collected from ground nuts in Benin and was used for several studies of aflatoxin production by *A. parasiticus* [30,31]. Spore stocks were maintained on potato dextrose agar (PDA, Difco, Becton, Dickinson, Sparks, MD, USA) and V8 (5% V8 juice 2% agar) plates.

### 3.2. RNA-seq Experiments

For RNA-seq studies *A. flavus* CA42, *A. flavus* AF70 and *A. parasiticus* BN9 were grown on PDA for 168, 40, and 40 h respectively. PolyA-mRNA was extracted from liquid nitrogen ground mycelia using a Dynabeads mRNA Direct Kit from Life Technologies [32]. cDNA libraries were prepared using the Ion Total RNA-seq Kit v2 from Life Technologies. Sequencing was done on an Ion Personal Genome Machine (Life Technologies). The RNA-seq data have been deposited at the National Center for Biotechnological Information (NCBI) Sequence Read Archive (SRA) with accession numbers of SRX470276 for *A. flavus* AF70, SRX470271 for *A. parasiticus* BN9 and SRX471362 for *A. flavus* CA42. The publicly available RNA-seq data for *A. oryzae* RIB40 (SRR610543) and *A. flavus* NRRL3357 (SRR610538) were obtained from the European Nucleotide Archive [33].

### 3.3. Databases Used for Annotation

Genome sequences and annotations for *A. flavus* NRRL3357 were acquired from NCBI [34]. Genome sequence for *A. oryzae* was acquired from AspGD [35]. Genome sequences for *A. parasiticus* and *A. flavus* AF70 were acquired from J. Craig Ventor Institute (JCVI) [36]. The RNA-seq data for all four organisms were mapped to the exons of each respective annotated genome using CLC Genomics Workbench, which calculated the RPKM (Reads Per Kilobase of exon model per Million mapped reads) value for each gene. The number of reads mapped to exons were 1.9, 2.9, 1.2, 0.7, and 1.2 million for *A. flavus* NRRL3357, *A. oryzae* RIB40, *A. flavus* AF70, *A. parasiticus* BN9, and *A. flavus* CA42, respectively. Domain predictions were done using the Conserved Domain Database (CDD) at NCBI [37].

## 4. Conclusions

The closely related *A. flavus*, *A. oryzae* and *A. parasiticus* genomes likely produce markedly different families of metabolites when grown on the same medium. These differences could help explain why *A. flavus* is more commonly associated with agricultural contamination events than is *A. parasiticus*.

It is generally supposed that ingestion of aflatoxins in cereal grains is responsible for the observed toxic effects caused by *A. flavus* on humans and animals [38,39]. That the *A. flavus* genome is able to encode enzymes that catalyze the production of non-aflatoxin toxic secondary metabolites indicates the importance of looking for additional toxins in contaminated cereal grains.

## Supplementary Files

Supplementary File 1 Supplementary Table 1 (XLSX, 73 KB)

Supplementary File 1 Supplementary Table 2 (XLSX, 19 KB)

Supplementary File 1 Supplementary Table 3 (XLSX, 11 KB)
